# Proteomic profiling of the mesenteric lymph after hemorrhagic shock: Differential gel electrophoresis and mass spectrometry analysis

**DOI:** 10.1186/1559-0275-8-1

**Published:** 2010-11-10

**Authors:** Ashley Zurawel, Ernest E Moore, Erik D Peltz, Janeen R Jordan, Sagar Damle, Monika Dzieciatkowska, Anirban Banerjee, Kirk C Hansen

**Affiliations:** 1 Proteomics Facility, University of Colorado School of Medicine, Aurora, USA; 2 Department of Pediatrics, University of Colorado School of Medicine, Aurora, USA; 3 Department of Surgery, Denver Health Medical Center, Denver, USA; 4 Department of Biochemistry and Molecular Genetics, University of Colorado School of Medicine, Aurora, USA

## Abstract

Experiments show that upon traumatic injury the composition of mesenteric lymph changes such that it initiates an immune response that can ultimately result in multiple organ dysfunction syndrome (MODS). To identify candidate protein mediators of this process we carried out a quantitative proteomic study on mesenteric lymph from a well characterized rat shock model. We analyzed three animals using analytical 2D differential gel electrophoresis. Intra-animal variation for the majority of protein spots was minor. Functional clustering of proteins revealed changes arising from several global classes that give novel insight into fundamental mechanisms of MODS. Mass spectrometry based proteomic analysis of proteins in mesenteric lymph can effectively be used to identify candidate mediators and loss of protective agents in shock models.

## Introduction

Multiple organ dysfunction syndrome (MODS) remains a leading cause of death due to trauma. Traumatic injury leads to systemic influx that precipitates post-traumatic organ dysfunction (liver, lungs, kidneys and heart) [1]. Previous work has demonstrated that postshock mesenteric lymph (PSML) serves as the conduit by which exudates are delivered to the systemic circulation [2,3]. Lymphatic diversion prior to trauma/hemorrhagic shock (T/HS) completely prevents or attenuates the shock induced lung injury, endothelial cell monolayer permeability, adhesion molecule expression and systemic neutrophil priming; further supporting the role of PSML as the mechanistic link between splanchnic ischemia reperfusion and remote organ dysfunction [2].

While it has been established that lymph serves as a conduit for the pathogenesis of T/HS-induced multiple organ failure, the specific mediators remain to be fully described. Lipid mediators involved in the priming of polymorphonuclear leukocytes (PMNs) for enhanced cytotoxicity and adherence have been suggested as important players in organ injury following hemorrhagic shock [3,4]. It is well known, however, that mesenteric lymph is the means of physiologic circulation of not only lipids, but also of proteins and of lipoproteins, and studies point to a significant difference in the concentrations of all three of these components between pre-shock and post-shock mesenteric lymph [5], suggesting synergistic interplay of these bio-molecules in mediating MODS. Additionally, Dayal *et al*. have demonstrated cytotoxicity in the aqueous fraction of PSML, possibly implicating proteins in the inflammatory processes leading to organ failure, and suggesting that characterizing the protein component of the lymph may provide key insights into postshock pathophysiology [6].

While recent studies have looked at the trauma patient plasma proteome [7], there are specific advantages of focusing our efforts on mesenteric lymph. During shock or stress blood circulation is drawn away from the gut area, to support the brain, heart and muscles. Upon resuscitation, the mesenteric lymph carries away the highly pro-inflammatory detritus from the hypo-perfused splanchnic bed, giving it a unique profile when compared to either plasma or serum samples [8]. The purpose of this study was to identify changes in post-shock mesenteric lymph from a well-studied animal model of T/HS. To accomplish this we utilize a differential gel electrophoresis (DIGE) approach. This involved labeling the pre- and post-shock samples with fluorescent dyes, two-dimensional gel electrophoresis for protein separation, followed by software analysis to identify significant changes, robotic spot extraction, in-gel proteolytic digestion and identification of proteins via tandem mass spectrometry analysis. Here, we measured the proteomic profile of mesenteric lymph to identify underlying processes involved in the disease physiology of shock.

## Results

### Differential comparison of pre and post hemorrhagic shock lymph in a rat model

**Figure 1:**
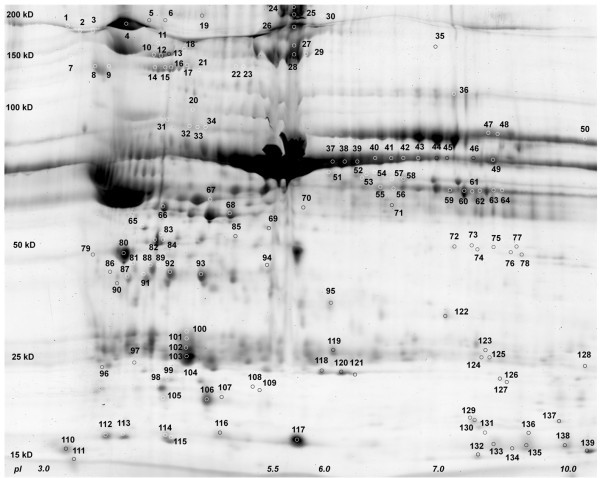
**Image of rat mesenteric lymph (collected with EDTA) separated by 2D gel electrophoresis**. Image of the Sypro stained preparative gel. The horizontal axis represent pH, here ranging from 3 on the left to 10 on the right, and the vertical axis represent molecular weight. A total of 500 μg of each pre and post-shock lymph, representing protein precipitated from a pool from three equally represented biological variants was focused onto a 24 cm Immobiline DryStrip, and then separated by molecular weight down the gel. Identifications made by DIGE and mass spectrometry analysis are marked by numbers that correspond to proteins listed in Table 1. (Attached File).

Three individual rats were used for lymph collection in the pre and post shock states. To identify candidate mediators and markers of MODS in the described trauma animal model we used DIGE to compare the protein content between pre- and post-shock mesenteric lymph, three analytical gels, one representing each individual animal, were run in technical duplicates. An internal standard approach was taken, using a pool of equal protein amounts of each sample, which allowed for the inter-comparison of the six gels. The internal standard was consistently Cy2 labeled, while samples were alternatively labeled with either Cy3 or Cy5 between the two sets of gels to control for potential dye-specific labeling artifacts. In addition, a preparative gel was run using a pool of lymph from the three animals, pre and post-shock, to facilitate protein identifications, and subsequently stained by Sypro protein stain and imaged (Figure 1).

One Cy2 image was selected as a reference gel, and gels were matched relative to this image. Following verification of alignment, 1853 spots were detected as consistently mapped to all gels. Of these 1853 spots, 467 had ANOVA (n = 6) p values < 0.05, and were further considered. 154 of the 467 significant spots also had ANOVA q values < 0.05, and these spots were selected to be excised, digested and identified by mass spectrometry. Along with the 154 spots, 12 additional spots were selected as prominent features of the gel, and were added to the list of potential proteins of interest. All 166 spots were automatically matched by the software to the Sypro stained image of the preparative gel.

Of the 166 spots excised, digested enzymatically with trypsin, and identified by mass spectrometry, 137 were confidently identified (Additional file 1: Table S1, Figure 1). Using fold change (from the fluorescent images) as a representation of relative protein abundance, 74 of the 137 identified proteins were seen to significantly (p < 0.05, q < 0.05 see methods) decrease following hemorrhagic shock in the described rat mode (Additional file 1: Table S1). Using the same standards of significance, 53 proteins significantly increased in the post-shock state, and while the remaining 12 proteins were not significantly up or down regulated, their identification contributes to the characterization of the overall hemorrhagic shock lymph proteome (Additional file 1: Table S1).

In addition, we attempted to use one of the analytical gels for protein identification to test our analytical platform. It was not expected that this would yield useful results however 78 out of 125 spots picked resulted in significant identifications and as a result will be included here (Additional file 2, Table S2). Using a similar image analysis approach as above, one Cy2 image was selected as a reference image, and all analytical gel images were matched relative to this one image. Once aligned, 1427 spots were consistently found across all gel images. Of these spots, 125 were selected to be picked based on their prominence on the Sypro stain of the analytical gel. Selected for land-marking purpose, these exploratory spots were intended to reflect a more or less random sampling, and not necessarily significant changes in either statistical measure or magnitude of volume fold change.

**Figure 2:**
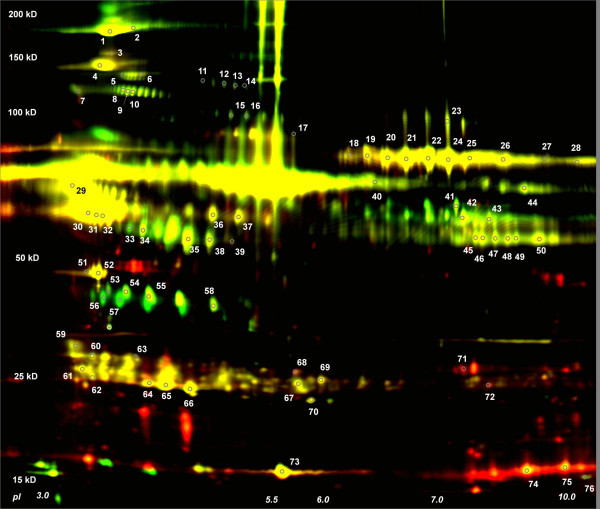
**Analytical DIGE Image (single animal) of rat mesenteric lymph**. Merged image of the Cy 5 and Cy3 scans from the analytical gel used for additional spot picking and protein identifications. The pH and MW range are the same as in Figure 1. 50 μg of pre and post-shock was used including a pooled internal standard labeled with Cy 2 that is not shown. Proteins identified are labeled with numbers that correspond to identification (Additional File 2, Table 2).

Of the picked and identified spots, 38 showed non-significant fold change (Additional file 2, Table S2, Figure 2). However, these identifications allow for a more comprehensive coverage of the mesenteric lymph proteome, as these features may have been overlooked under the more stringent selection conditions used for the preparative gel analysis. Along with these 38 proteins, 29 identified proteins were significantly up-regulated and 9 significantly down-regulated according to the previous parameters of analysis which included the analytical and preparative gels (Additional file 2, Table S2, Figure 2).

### Loss of Anti-Proteases

From the identifications made from the preparative gel, certain proteins surface as relevant to post shock physiology. The anti-proteases inter-α-inhibitor H3, inter-α-inhibitor H4 and α-1-macroglobulin were found in multiple spots decreasing in abundance. The identification of inter-α-inhibitor H3 was made in seven total spots. Three of these identifications were made at approximately 180 kD and within a pI range of 3.5 to 4.5 (Additional file 1: Table S1). Two of the seven identifications were made from spots picked at approximately 50 kD lower in MW and within the same pI range. The final two identifications were made, one in the 180 kD range but at a significantly higher pI of about 5.5, and another at approximately 100 kD in the pI range of 5.0. The fold change of all seven identifications varied from depletion between 1.52 to 1.79 fold, with no discernable distribution pattern between fold change and molecular weight or isoelectric point (Additional file 1: Table S1). Species-specific Uniprot database information for inter-α- inhibitor 3 indicates that the expected molecular weight of this protein is 100 kD and the expected isoelctric point is 5.85; the observed experimental aberrances suggest alteration to the parent protein by post-translational modification or alternative splicing.

Similarly, inter-α-inhibitor H4 is seen to be depleted. Inter-α-inhibitor H4 was identified in four spots, all around a molecular weight of 150 kDa, a pI of approximately 4.0. The fold change of this protein's depletion in post-shock lymph varies little, in a range between 1.76 and 1.91. The expected molecular weight of inter-α-inhibitor H4 is approximately 100 kDa, and its expected pI is 6.5 (Additional file 1: Table S1). The higher experimental molecular weight and lower experimental isoelectric point again points to possible protein modifications.

The multiple identifications of the anti-protease α-1-macroglobulin is a case where dynamic protein changes are evident. According to its Uniprot database entry, it should migrate to approximately 170 kDa at an isoelectric point of 6.46. In this study, α-1-macroglobulin was identified eleven times, at various molecular weights and pIs. Three identifications were made near 170 kDa, but were seen at pIs between 3.0 and 4.0. Six identifications were made near 40 kDa, in a similar pI range, with the exception of one of these identifications being made at a pI approaching 5.5. All above listed α-1-macroglobulin identifications decrease in relative abundance in PSML, varying in range between 1.70 and 3.55 fold. The two remaining identifications were made at lower molecular weights: one at approximately 25 kDa and at a pI of almost 7.0, and the other closer to 20 kDa and at a pI near 5.0 (Additional file 1: Table S1). Notably, these two identifications increased in abundance (by 2.09 fold and 3.45 fold respectively).

### Intracellular Proteins

The intracellular enzymes parvalbumin-α, β-enolase and aldolase were identified. The identification of intracellular enzymes in PSML suggests tissue injury. All identifications for these proteins were seen in spots that increased in protein abundance relative to the pre-shock lymph. Two isoforms of aldolase were identified: fructose-bisphosphate aldolase A, and fructose-bisphosphate aldolase B. Aldolase A was identified three times, all within a few kD of the expected molecular weight of 40 kD, and at approximately the expected pI of 8.0. Similarly, aldolase B was identified four times, and was found at approximately the expected molecular weight and pI for this isoform.

### Coagulation related

Hemolysis, blood coagulation and fibrinolysis are integral mechanisms of the trauma-induced physiologic response and pre-dispose a patient to sepsis [9]. Fibrinogen exists as a heterohexamer linked by disulfide bonds, composed of 2 sets of 3 non-identical chains: alpha, beta, and gamma [10]. All three subunits decreased in abundance in PSML, however, discrepancies between both molecular weight and isoelectric point are noted as may be expected for a protein with known activation cleavage sites. The alpha subunit of fibrinogen was identified five times as a protein that decreased and once as a protein that increased, notably at consistently lower molecular weights and slightly higher isoelectric points than expected for the unprocessed, full-length protein. The beta subunit was identified four times, within a few kilodaltons of the expected 60 kDa and hovering around the expect pI of 7.6. Similarly, the gamma chain was found twice, close to the expected 50 kDa and pI of 5.6. (Additional file 1: Table S1). Fibrinogen alpha and beta are cleaved when triggered by thrombin into thrombopeptide A and B, uncovering the N-terminal polymerization sites on the α and β chains, allowing them to interact with the C-terminal γ sites, and be cross-linked by factor XIIa, resulting in clot formation [11]. The noted lower molecular weights and higher pIs of the identified alpha subunits may be indicative of such dynamic interplay; however, it is noteworthy that both the beta and gamma chains remain consistent with their expected electrophoresis properties, suggesting that these identified forms remained largely intact.

Lysis of red blood cells in the post-shock state are illustrated by an increase in both the α and β chains of hemoglobin concurrent with the identification of haptoglobin, shown to decrease in PSML. As haptoglobin is involved with hemoglobin degradation and in concert this process works to prevent damage due to iron toxicity, this shift suggests biological relevance. Transferrin, another iron-binding protein, was identified eight times, with an overall decreasing trend in post-shock lymph (six of the eight identifications were made from significantly decreasing spots; one of the two identifications that had an increasing abundance in post-shock lymph was made at a MW lower than 15 kDa, suggesting a fragment from its 76 kDa precursor (Additional file 1: Table S1)). Similarly, ceruloplasmin was twice identified as decreasing in PSML. Ceruloplasmin is involved in iron transport across cells, and is involved in many cellular processes including iron metabolism [12]. Its lowered abundance further points to the potential involvement of endothelial cell damage during shock induced injury as a result of heme-generated/propagated reactive oxygen species [13].

The identifications made from the analytical gel were, on a global level, consistent with those made from the preparative gel. A depletion of proteases such as inter-α-inhibitor H3 and H4 and α-1-macroglobulin were consistent with the preparative gel (Additional File 2, Table S2). Intra-cellular enzymes indicative of tissue injury were not identified as seen in the preparative gel. However, both coagulation and plasma proteins such transferrin and ceruloplasmin were seen to decrease in the post-shock state, consistent with the afore-mentioned trend observed from the preparative gel (Additional File 2, Table S2).

### Western Blot

**Figure 3:**
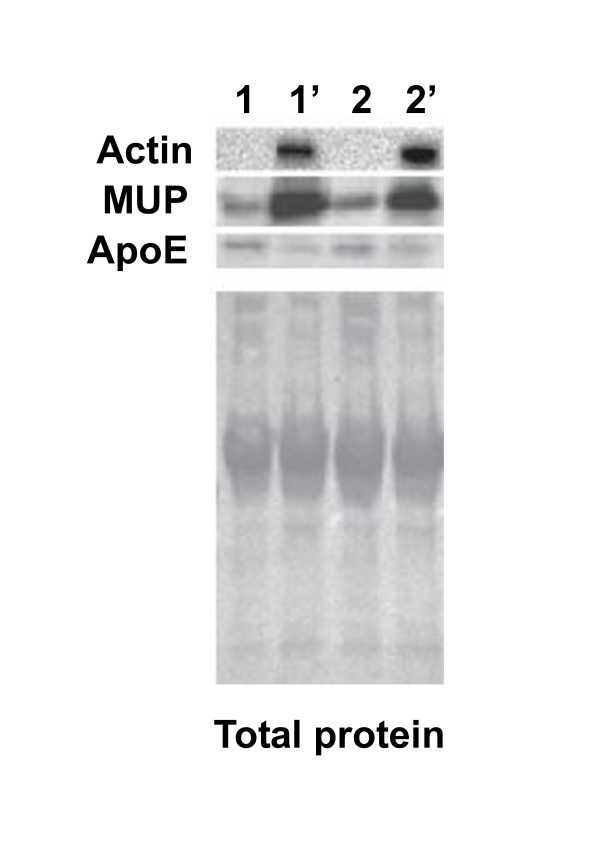
**Western blots of mesenteric lymph before and after shock**. The expression of β-actin, major urinary protein (MUP) and apolipoprotein E (Apo E) in pre-shock and post shock mesenteric lymph. Lanes 1 and 2 are pre-shock meserteric lymph form two animals; the lanes 1' and 2' are from post-shock mesenteric lymph from the same animals. Each lane contained 20 μg total protein. Ponceau S staining (lower image) of the membrane was used to evaluate protein loading and transfer.

To validate our proteomic results, we measured protein levels of three selected targets of interest in mesenteric lymph by Western blotting. Consistent with our proteomic results, Western analysis confirmed increased protein levels of β-actin, major urinary protein, and decreased levels of apolipoprotein E (Figure 3.) in post shock mesenteric lymph as compared with preshock lymph.

## Discussion

This study aimed to characterize the dynamic changes in the protein fraction of lymph after hermorrhagic shock followed by resuscitation. It has been well established that mesenteric lymph serves as a mechanistic conduit during hemorrhagic shock, and it has also been shown that the protein fraction of lymph is at least in part responsible for its pathophysiology [6]. Previous studies have used two-dimensional gel electrophoresis and mass spectrometry methods to analyze the plasma proteome of trauma patients [7] and the protein fraction of lymph itself [14]. In this study we aimed to characterize the protein fraction of mesenteric lymph in the context of a hemorrhagic shock model. Our proteomic results point to several potential mechanistically relevant roles of mesenteric lymph in the progression of T/HS as suggested by the identification of numerous proteins that either increase or decrease in the post-shock state.

The DIGE technique employed in this work has the distinct advantages over non-2D gel proteomic approaches in that protein isoforms can be separated if they differ sufficiently by mass or charge. There are numerous examples in our dataset of apparent molecular weight discrepancies with the reported full length protein. This provides the opportunity to further define the protein present. However, some identifications arise from low sequence coverage making conclusions about isoforms challenging and observation of posttranslational modifications rare. In addition, the advantage is somewhat offset by the observation that only more abundant proteins are identified.

Recent work has begun to investigate how a few proteins, namely albumin, factor into the physiological effect of lymph during shock [15,16]. Recently Kaiser *et al*. showed that the N-terminal 24 amino acids peptide of the albumin was significantly increased in post-T/HS lymph collected from animals. In this study we identified albumin containing gel spots with apparent molecular weights of 20 and 25 kDa (e.g., preparative gel spots 99, 104, 109). One example was spot 104, identified with high sequence coverage from peptides between residues 29-217 consistent with increased proteolytic processing of albumin in post shock lymph.

The overall decreasing trend in coagulation proteins in the post-shock state is consistent with the noted coagulopathy observed in hemorrhagic shock patients [9,17]. Its systemic activation results in the activation of immune mechanisms that can lead to increased vascular injury [18]. While the collection method using anti-coagulant may not be a means to correct for all sample-dependent coagulation, the link between coagulopathy and traumatic injury is well represented in the data set. The noted decrease in protease inhibitors is of interest. Inter-alpha-inhibitor H4 and H3, for example, have been shown to reduce complement-dependent lung injury *in vivo *[19], suggesting that their decrease could be a contributing factor to the hemorrhagic state. In addition to protein level decreases the dilution of body fluids that accompanies major resuscitation efforts will further lower the concentration of anti-proteases. Based on the appearance of increased anti-protease fragments (e.g., spots 105, 107, and 108) it would appear that this class of proteins are being consumed and potentially tipping the protease/anti-protease balance. Finally, the finding of intracellular enzymes such as the A and B isoforms of aldolase, a glycolytic enzyme with actin-binding properties [20] may be mechanistically relevant to injury-related biological processes, such as lung injury, a process dependent on cytoskeletal rearrangements [21].

Several differences in the trauma proteome between pre- and post-shock states were identified; many are unique candidates for active contributors to the generation of MODS. Many of the proteins identified deviated from the expected molecular weight and isoelectric point and were identified in multiple locations on the gel indicating distinct protein isoforms for further study. Overall, a decrease in coagulation-associated proteins, the depletion of protease inhibitors, and an observed increase in intracellular proteins indicative of injury on a global level provide a schematic view of how proteins in the mesenteric lymph change upon traumatic injury. Future studies will validate if these identified changes play a functional role in the onset of MODS.

## Methods

All animal experiments were performed in accordance with protocols approved by the Institutional Animal Care and Use Committee at the University of Colorado Denver.

Pentobartial sodium was purchased from Abbott Labs (North Chicago, IL). Polyethylene tubing was purchased from Intrametic, Fisher Scientific. Heparin was purchased from American Pharmaceutical Partner, In (Schaumburg, IL). DIGE experiment reagents were purchased from GE healthcare. All other reagents were purchased from Sigma-Aldrich Corp. (St. Louis, MO) unless otherwise specified.

### Hemorrhagic shock

Controlled hemorrhage was induced to male Sprague-Dawley rats weighing 218 mg to 351 mg (Colorado State University) that had been housed in climate controlled barrier facility with 12 hr light/dark cycles with free access to food and water. The animals were anesthetized with 50 mg/kg pentobarbital sodium via intraperitoneal injection. The femoral artery and vein were then cannulated with polyethylene (PE) 50 tubing and the blood pressure and mean arterial pressure were monitored using a ProPaq invasive monitoring device (Welch Allyn Inc., Skaneateles Falls, NY). A separate skin puncture was created to tunnel the catheters prior to closure of the groin incision. A 3 cm midline laparotomy was performed. The bowel was eviscerated and rotated to the left, and the mesenteric duct and accessory duct (located adjacent to the superior mesenteric artery) were isolated by blunt dissection. The main lymphatic duct was cannulated with PE 100 tubing and secured with 7-0 prolene suture. The accessory duct was then ligated with suture, and the catheter was tunneled posteriorly through the skin. The laparotomy incision was closed in a two layer fashion and lymph collection took place in half-hour intervals into 1.6 mL tubes containing 1.0 mg ethylenediaminetetraacetic acid (EDTA) followed by rapid freezing in liquid nitrogen. After 1 hour of lymph collection, hemorrhagic shock was induced by controlled blood loss to maintain a mean arterial pressure (MAP) of 30 mmHg and sustained for 40 min. Euthermic body temperature was maintain with a heat lamp and monitored rectally in regular intervals. Resuscitation was performed by infusing 2x shed blood volume in normal saline over 30 min, followed by 1/2 shed blood volume returned over 30 min, then completed with 2x shed blood volume in normal saline over 60 min. Lymph collection continued for one hour post completion of resuscitation and all lymph samples were then centrifuged at 5000 × *g *for 10 min to remove cellular components. The lymph supernatant was collected and frozen in liquid nitrogen, and all lymph samples were stored at -80°C until processing. The fractions collected between 2-3 hours following resuscitation were consistently bioactive by a number of priming, signaling and physiological tests [22]. Protein quantification was performed using the BCA protein assay kit (with BSA as standard) to create a regression analysis to estimate overall protein concentration for each hourly sample [23]. In general post-shock mesenteric lymph was approximately 1/5^th ^as concentrated as pre-shock lymph.

### Lymph Sample Preparation and Protein Isolation

Lymph samples collected with EDTA were methanol-chloroform precipitated [24] and the resulting protein pellet was re-suspended in rehydration buffer at room-temperature overnight [25]. For preparative gel analysis, equal protein weights of lymph from three animals were pooled prior to precipitation. A small aliquot of lymph at each time point was kept unprecipitated. Protein concentration was quantified using the Bradford assay as previously described [26].

### Cy Dye Labeling and 2D Electrophoresis

A pooled internal standard approach was used, and two sets of analytical technical replicates were run, each representing an individual rat [27]. An equal fraction from each animal of 500 μg total protein was combined, aliquoted, frozen with LN_2_, and kept at -80°C until used, providing an internal standard for all subsequent 2D gel experiments. Each analytical gel represents one animal differentially comparing the pre (initial collection) and post shock (3 hours from the start of resuscitation) states. The pooled internal standard was consistently Cy2 labeled; individual samples were alternatively labeled with Cy3 and Cy5 dyes between technical runs to control for any dye-specific labeling artifacts. Along with the second set of analytical gels, a preparative gel was run, consisting of 500 μg of a pre-shock protein pool and 500 μg of a post-shock protein pool made with equal protein amounts from lymph collected with EDTA from all three animals, along with the 50 μg Cy2 labeled internal standard.

All Cy labeling was done according to the manufacturer's protocol, where 200 pmol of dye was used to label 50 μg of protein (Cy dyes DIGE Fluors, GE Healthcare, Piscataway, NJ), under standard minimal dye labeling conditions [28].

Each set of analytical samples were passively rehydrated into Immobiline DryStrips 24 cm pH3-10 (GE Healthcare) overnight or for at least 18 hours, followed by isoelectric focusing using an IPGphor IEF unit (Amersham Biosciences/GE Healthcare). Focusing was performed at 20°C, at 50 μA per strip, according to the following step and hold sequence: 1) 500 V for 500 Vhr, 2) 1000 V for 1000 Vhr, 3) 8000 V for 24 000 Vhr, 4) 8000 V for 64 000 Vhr and 5) 8000 V for 64 000 Vhr.

For the preparative gel, labeling and rehydration was performed as it was with the analytical gels, with the exception that after the labeling step, 450 μg of each sample was added. The focusing parameters were the same, and included the following step and hold voltages: 1) 250 V for 1000 Vhr, 2) 500 V for 1000 Vhr, 3) 1000 V for 1000 Vhr, 4) 8000 V for 66 000 Vhr, 5) 8000 V for 66 000 Vhr and 6) 8000 V for 66 000 Vhr.

After focusing and prior to eletrophoresis, each strip was incubated at room temperature for 15 hours in reducing and alkylating solutions as previously described [29]. Strips were then loaded onto second dimension 9-16% tris-glycine gels (Jules Gels, Milford, CT), sealed with agarose (SDS equilibrium buffer, 0.5% (w/v) agarose, and 0.25% (v/v) of saturated aqueous bromophenol blue) and run at 20 W per gel on the Ettan Dalt System (Amersham/GE Healthcare) for approximately 4 to 6 hours.

### Gel Imaging

Imaging was done on a Typhoon 9400 Variable Mode Laser Imager (Amersham/GE Healthcare) [30]. The gels that were used for protein identification were then fixed for 1 hour in 7.5% acetic acid/10% methanol, and stained overnight with Sypro Ruby protein gel stain (Invitrogen/Molecular Probes, Eugene, OR). Following destaining (7.5% acetic acid/30% methanol), gels were re-imaged at 100 μm resolution (laser excitation 532 nm, emission 560 nm, LP Gen. Purple).

### Gel image analysis

Images were analyzed using Progenesis SameSpots v 3.1 (Nonlinear Dynamics, Durham, NC) software. One Cy2 image was selected as the reference image, and all gels were mapped to this reference image. Approximately 20 vectors were hand-placed on each additional gel image to facilitate the gel-to-gel matching; afterwards, automatic software matching was performed. Alignment was verified manually, matching artifacts deleted, and misalignments corrected. Following alignment, statistical analysis was performed, using normalized volume as a representation of protein abundance. Resulting ANOVA p and q values were used to assign statistical significance to detected changes in the pre and post states; both were limited to values < 0.05. The corresponding spots were then matched to a Sypro stained image of the preparative gel, which was first mapped to the reference image (Figure 1, Additional file 1: Table S1).

In addition, one set of analytical gels were analyzed independently, and a preliminary set of spots were selected to be picked on one individual replicate gel (animal R32) based on visual inspection and basing picks on viewed changes and spot abundance (Figure 3, Additional file 1: Table S1).

### Spot Picking and Tryptic Digestion

Proteins of interest were excised from the two gels using an robotic spot picker (Ettan SpotPicker software v 1.10, GE Healthcare/Amersham Bioscience) fitted with a 1.0 mm deep, 1.4 mm in diameter picker head, and placed in 96 well plates, which were then transferred to an Ettan Digester (software v 1.10, GE Healthcare/Amersham Bioscience). Excised spots were washed twice with 100 μL of 50 mM ammnonium bicarbonate, once with 100 μL of 75% acetonitrile and once with 100 μL of 100% acetonitrile and left to dry at room temperature. Sequencing grade modified trypsin in 25 mM ammonium bicarbonate (1:4 v/v; Promega, Madison, WI) was added to each gel plug, plates were sealed and after a 30 minute incubation at 4°C were left at room temperature overnight for digestion. Following digestion, the peptides were extracted with 1.0% FA solution and then again with 50% ACN and 1.0% FA.

### Mass Spectrometry

Matrix-assisted laser desorption ionization (MALDI) tandem time-of-flight (TOF/TOF) mass spectrometry was carried out on an Applied Biosystems 4700 mass spectrometer, or an Applied Biosystems 4800 mass spectrometer. A saturated solution of alpha-cyano-4-hydroxycinnamic acid was prepared in acetonitrile/water (0.1% TFA). The equal parts of sample and α-cyano-4-hydroxycinnamic acid (7%) were manually spotted onto 100 well and/or 384 well stainless steel target plates (Applied Biosystems, Foster City, CA) and allowed to air dry prior to insertion into the mass spectrometer. Mass spectra were obtained for mass range from 800 to 4000 Daltons in reflector mode. All spectra were processed in Data Explorer v 5.0 (Applied Biosystems), and internally calibrated to a minimum of three monoisotopic trypsin autolysis peptides. Spectra were then used to interrogate sequences in the Swiss-Prot database using Mascot Daemon software v 2.2.2 (Matrix Science, Boston, MA) running the Mascot server (V 2.2). The search parameters were as follows: mass tolerance 100 ppm, *Rattus *taxon, enzyme specificity to trypsin and one missed cleavage. Trypsin specificity was used allowing for 1 missed cleavage. The modifications of Met oxidation, protein N-terminal acetylation, peptide N-terminal pyroglutamic acid formation were allowed for (used for all searches below).

Nano-liquid chromatography tandem mass spectrometry analysis was performed using an LTQ-XL Linear Ion Trap Mass Spectrometer or an LTQ-FT Ultra Hybrid ion cyclotron resonance mass spectrometer (ThermoFisher; San Jose, CA).

2 μL of tryptic digest sample was injected onto a reverse-phase column using a cooled (9°C) autosampler (Eksigent; Dublin, CA) connected to a HPLC system run at 120 μL/min before the T-split and ~400 nL/min post-split (Aligent; Santa Clara, CA). The column was made from an in-house pulled 100 μm i.d. × 150 mm fused silica capillary packed with Jupiter C_18 _resin (Phenomex; Torrance, CA) kept at a constant 40°C using an in-house built column heater [31]. A gradient of 12% to 30% of ACN over a sixty minute run was employed for peptide separation. The column effluent was coupled directly to a LTQ-XL Linear Ion Trap mass spectrometer with an in-house built nanospray ion source. Data acquisition was performed using the instrument supplied Xcalibur (version 2.0.6) software. The sixty minute LC runs were monitored by sequentially recording the precursor scan (MS) followed by three collision-induced dissociation (CID) acquisitions (MS/MS). Normalized collision energies were employed using helium as the collision gas.

In addition, samples were analyzed on a LTQ-FT hybrid mass spectrometer. Peptide desalting and separation was achieved using a dual capillary/nano pump HPLC system (Agilent 1200, Palo Alto, CA). On this system 8 μL of sample was loaded onto a trapping column (ZORBAX 300SB-C_18_, dimensions 5 μm i.d. × 5 mm, Agilent Technologies, Santa Clara, CA) and washed with 5% ACN, 0.1% FA at a flow rate of 15 μL/min for 5 minutes. At this time the trapping column was put online with the nano-pump at a flow rate of 350 nL/min. An 85 minute gradient of 8 - 40% ACN was used to separate the tryptic peptides on an in house packed column. Data acquisition and analysis was performed as described above with the following modifications: for every MS scan four CID-induced MS/MS scans were acquired; MS mass tolerance was set to +/- 10 ppm for precursors; and +/- 0.6 Da for MS/MS fragment ions.

An in-house script was used to create de-isotoped centroided peak lists from the raw spectra (.mgf format). These peak lists were then interrogated against all rodent entries in the Swiss-Prot database using Mascot Daemon software v 2.2.2 (Matrix Science, London, UK) using an in-house Mascot server (v 2.2). Mass tolerances were +/- 1.2 Da for precursor ions, and +/- 0.6 Da for MS/MS fragment ions for spectra acquire from the LTQ-XL; +/- 10 ppm for MS peaks, and +/- 0.6 Da for MS/MS fragment ions for spectra acquired from the FT-ICR.

### Western Blot Analysis

Proteins (approximately 20 μg per lane for lymph samples) were separated by 1D SDS-PAGE on 4-20% bisacrylamide gel and transferred electrophoretically to a nitrocellulose membrane. The filters were stained with 1% Ponceau S in 5% acetic acid to confirm proper transfer. For destaining, the blot was washed with alkaline water. Blocking was performed for 1 hour at room temperature in 5% nonfat dried milk, in 100 mM PBS. Incubation with antibodies to MUP (Santa Cruz Biotechnologies Inc., Santa Cruz, CA, Cat. # R-181), Apo E (Santa Cruz Biotechnologies Inc., Santa Cruz, CA, Cat. # R-20) or β-actin (Cell Signaling Technologies Inc., Danvers, MA, Cat. #4967) were performed overnight at 4°C in 5% nonfat dried milk in 100 mM PBS containing 0.5% Tween 20. Bands were detected with goat anti-rabbit (Thermo Scientific, Rockford, IL, Cat. # 31460) or goat anti-mouse horseradish peroxidase (Thermo Scientific, Rockford, IL, Cat. #31430) using West Pico enhanced chemiluminescence kit (Thermo Scientific, Rockford, IL), and visualized with the ChemiDoc XRS gel documentation system (Bio-Rad, Hercules, CA). Quantification of band intensities was performed with Quantity One analysis software (Bio-Rad, Hercules, CA).

## List of Abbreviations

DB: Database; DIGE: Differential in-gel electrophoresis; ESI: Electrospray ionization; nLC: Nano-flow liquid chromatography; LTQ: Linear ion trap mass spectrometer; MS: Mass spectrometry; MS/MS: Tandem mass spectrometry; RP: Reversed Phase; FT-ICR: Fourier Transformed Ion Cyclotron Resonance mass spectrometer.

## Authors' contributions

EM, EP, JJ, FG and SD carried out animal model surgery and sample collection. AZ carried out the sample labeling and 2D-DIGE analysis. KH, AZ and MD performed mass spectrometry data collection and data analysis. KH, EP, AZ, EM, MD and AB drafted the manuscript. FG carried out the western analysis. KH, AB and EM conceived of the study, and participated in its design and coordination. All authors read and approved the final manuscript.

**Financial Support**: This work was supported in part by grants from the National Institutes of Health, National Institute of General Medical Sciences (T32-GM008315 and P50-GM049222), National Center for Research Resources (S10RR023015), and University of Colorado Comprehensive Cancer Center Core Support (P30 CA046934-17).

## Supplementary Material

Additional File 1 **Table S1: Preparative Gel Protein Identifications**. Click here for file

Additional File 2 **Table S2: Analytical Gel Protein Identifications**. Click here for file
